# Early breastfeeding practices: Descriptive analysis of recent Demographic and Health Surveys

**DOI:** 10.1111/mcn.12535

**Published:** 2017-10-16

**Authors:** Laura Oakley, Lenka Benova, David Macleod, Caroline A. Lynch, Oona M. R. Campbell

**Affiliations:** ^1^ Faculty of Epidemiology and Population Health London School of Hygiene and Tropical Medicine London UK

**Keywords:** birth, breastfeeding, breastfeeding initiation, inequalities, low income countries, maternal public health

## Abstract

The aim of this study was to describe early breastfeeding practices (initiation within 1 hr of birth, no prelacteal feeding, and a combination of both—“optimal” early breastfeeding) according to childbirth location in low‐ and middle‐income countries. Using data from the most recent Demographic and Health Survey (2000–2013) for 57 countries, we extracted information on the most recent birth for women aged 15–49 with a live birth in the preceding 24 months. Childbirth setting was self‐reported by location (home or facility) and subtype (home delivery with or without a skilled birth attendant; public or private facility). We produced overall world and four region‐level summary statistics by applying national population adjusted survey weights. Overall, 39% of children were breastfed within 1 hr of birth (region range 31–60%), 49% received no prelacteal feeding (41–65%), and 28% benefited from optimal early breastfeeding (21–46%). In South/Southeast Asia and Sub‐Saharan Africa, early breastfeeding outcomes were more favourable for facility births compared to home births; trends were less consistent in Latin America and Middle East/Europe. Among home deliveries, there was a higher prevalence of positive breastfeeding practices for births with a skilled birth attendant across all regions other than Latin America. For facility births, breastfeeding practices were more favourable among those taking place in the public sector. This study is the most comprehensive assessment to date of early breastfeeding practices by childbirth location. Our results suggest that skilled delivery care—particularly care delivered in public sector facilities—appears positively correlated with favourable breastfeeding practices.

## 1 INTRODUCTION

Breastfeeding has numerous benefits for both mothers and children, and offers crucial protection against infectious morbidity and mortality in children (Victora et al., 2016). Scaling up breastfeeding to near universal levels could prevent up to an estimated 13.8% of deaths in children younger than 24 months globally each year (Black et al., 2013; Victora et al., 2016). The burden of suboptimal breastfeeding disproportionately affects low‐and middle‐income countries (Unicef, 2016); in the 2010 Global Burden of Disease Study, suboptimal breastfeeding was one of the three leading causes of disease across much of Sub‐Saharan Africa (Lim et al., 2012). Although many low‐ and middle‐income countries (LMICs) have strong breastfeeding traditions, adherence to World Health Organization (WHO) Infant and Young Child Feeding recommendations is poor, particularly in terms of timely initiation and breastfeeding exclusivity. Only one half of infants born in LMICs are put to the breast within an hour of birth as recommended by WHO (Victora et al., 2016). Delayed breastfeeding initiation increases the risk of neonatal mortality (Debes, Kohli, Walker, Edmond, & Mullany, 2013; Neovita Study Group, 2016), and is associated with a higher probability of prelacteal feeding (defined as giving any food or liquid other than breastmilk before the initiation or establishment of breastfeeding) and the withholding of colostrum (concentrated first breastmilk rich in immune and growth factors). The provision of prelacteal feeds, often for ritual reasons, is prevalent in many LMICs (Akuse & Obinya, 2002; Chandrashekhar et al., 2007; Engebretsen et al., 2007; Khanal, Adhikari, Sauer, & Zhao, 2013; Patel, Banerjee, & Kaletwad, 2013; Shirima, Greiner, Kylberg, & Gebre‐Medhin, 2001). Both prelacteal feeding and the withholding of colostrum are known to have an adverse effect on later breastfeeding practices (Patil et al., 2015; Sundaram et al., 2013).

The intrapartum and immediate post‐natal period is a crucial window of opportunity for promoting optimal breastfeeding practices. Childbirth care is likely to affect breastfeeding practices through the provision of appropriate advice and support, and through the facilitation of practices known to be conducive to breastfeeding (e.g., skin‐to‐skin contact immediately after birth; Moore, Anderson, Bergman, & Dowswell, 2012). Any potential effect of delivery care on breastfeeding practices is most likely to be observed for early breastfeeding outcomes.

Place of childbirth has been linked to quality of care indicators among which is optimal breastfeeding (Fink, Ross, & Hill, 2015). A small number of studies have investigated childbirth location as a potential determinant of breastfeeding practices, commonly using a simple dichotomous categorisation of home or facility birth. There is some evidence from recent studies that early initiation of breastfeeding is more common among births in health facilities (Kimani‐Murage et al., 2011; Ogunlesi, 2010; Senarath et al., 2012), although this finding is not consistent across all studies. It is notable that very few of these studies differentiate between sector of facility (private or public) and whether the birth was attended by a skilled attendant. Improved understanding of trends in breastfeeding behaviour according to delivery setting should help inform strategies for promoting optimal breastfeeding.

The objective of this study was to comprehensively describe early breastfeeding practices by childbirth setting and subtype using data from Demographic and Health Surveys (DHS) across 57 low‐ and middle‐income countries.

**Key messages**

- Only 28% of infants in low‐ and middle‐income countries (LMICs) benefit from optimal early breastfeeding, with wide variation by region and childbirth location.
- Cross‐sectoral comparisons suggest that early breastfeeding outcomes are more favourable among facility births compared to births at home in LMICs, particularly in Sub‐Saharan Africa and South/Southeast Asia.
- Among home births, positive early breastfeeding practices are generally higher among births attended by a skilled birth attendant.
- Across all four LMIC regions, early breastfeeding practices are more favourable in births in public sector facilities compared to private sector births.

## 2 METHODS

### 2.1 Data

DHS are population‐based cross‐sectional household surveys which use a core set of questionnaires tailored to country setting (Fabic, Choi, & Bird, 2012). We included the most recent survey for all countries that had a DHS between 2000 and mid‐2013. The resulting dataset contained 57 countries (Supplementary Table S1) from four geographic regions: Sub‐Saharan Africa (30 countries), North Africa/West Asia/Europe (nine countries), South/Southeast Asia (10 countries), and Latin America and the Caribbean (eight countries). The regions were constructed based on a classification of countries by Measure DHS, following other analyses of DHS data (Montagu, Yamey, Visconti, Harding, & Yoong, 2011). For simplicity, we refer to these regions as Sub‐Saharan Africa, Middle East/Europe, South/Southeast Asia, and Latin America in this paper. Data about breastfeeding and location of childbirth are based on women's self‐reports.

The DHS received institutional review centrally (ICF International), and approval by every participating country. This study was approved by the Research Ethics Committee of the London School of Hygiene and Tropical Medicine, UK.

### 2.2 Population

Breastfeeding indicators and childbirth location for the most recent live birth were examined among women aged 15–49 with a birth in the 24 months before the survey. We restricted the sample to children who survived to at least 1 month in an attempt to exclude infants who were less likely to breastfeed, perhaps because of separation from their mother or inability to breastfeed due to prematurity or poor health condition at birth. Childbirth setting may also differ for these children, for example, mothers who went into labour early may be more likely to seek facility care.

### 2.3 Indicators and definitions

#### 2.3.1 Breastfeeding

Our outcomes of interest were key infant feeding indicators reflecting breastfeeding practices in early life. We included two WHO‐recommended indicators for assessing infant and young child feeding practices: the proportion of children ever‐breastfed, and the proportion of children who were put to the breast within 1 hr of birth (World Health Organization, 2008). We derived two additional indicators from the data. Firstly, the proportion of children who received breastmilk only in the first 3 days (no “prelacteal feeding”), derived from a negative response to the question “In the first three days after delivery, was [child's name] given anything to drink other than breast milk?”. The second additional indicator was the proportion of children who were fed “optimally” in the in the early days after birth (put to the breast within 1 hr and no prelacteal feeding). Not all countries collected all the data that was necessary to construct the four breastfeeding indicators. A full description of the indicators and denominators is presented in Table 1.

**Table 1:** Infant and young child feeding (IYCF) indicators used in the study

Indicator	WHO IYCF indicator (World Health Organization, 2008)	Description	Denominator	Included countries
Ever breastfed	Optional indicator 9	% breastfed at least once	Most recently born children aged <24 months at interview and alive at >1 month	Available for all 57 countries
Early breastfeeding	Core indicator 1	% put to the breast within an hour of birth		Available for all 57 countries
No prelacteal feeding	n/a	% not receiving anything other than breastmilk in first 3 days		Not available: Vietnam
Optimal early breastfeeding	n/a	% put to the breast within an hour of birth and not receiving anything other than breastmilk in first 3 days		Not available: Vietnam

#### 2.3.2 Childbirth location and attendant

Women were asked for the location of their most recent live birth in the recall period. We first characterised childbirth locations as home or facility‐based. Among facility deliveries, we further differentiated locations as being public‐ or private‐sector. Public‐sector childbirth locations were those occurring in public, government or social security health facilities. Private‐sector locations were those occurring in facilities outside the public sector, such as in private facilities, private health professional locations, faith‐based organisation facilities, non‐governmental organisation facilities, and other private facilities, as previously described (Benova et al., 2015). Respondents were asked to list all people who assisted with the delivery; we considered the person with the highest level of qualification, and classified home‐based births as having been attended by a skilled birth attendant (SBA) or not, according to country‐level criteria (Benova et al., 2015; Footman et al., 2015).

### 2.4 Missing data

Analyses were conducted on the 99.5% of births in the sample that had nonmissing values for the two main variables surrounding childbirth (childbirth location—including sector of childbirth facility and delivery attendant). Where information was missing on breastfeeding indicators, we assumed the infant was not fed optimally (ever breastfeeding was missing for 0.3% of observations, early breastfeeding 0.1%, and pre‐lacteal feeding 2.7%).

### 2.5 Analysis and construction of regional and overall summary measures

All analyses were conducted using Stata 13. DHS surveys are conducted using a multistage cluster sampling strategy, and women in each DHS survey have an individual sample weight that is used to calculate country‐level representative summary statistics. We used these and further produced region‐level and overall summary statistics by applying weights that accounted for both country‐specific survey design and national population size (so e.g., India contributed more to overall and South/Southeast Asia estimates than Nepal), to ensure that estimates are representative of the population residing in study countries/regions. To capture the extent of variability across the included countries, we report ranges and medians. Where overall percentages for all regions are reported in the text, these are population‐weighted means of all included countries unless stated otherwise. We present differences in breastfeeding outcomes for home vs. facility‐based deliveries by country, but differences for SBA vs. non‐SBA and public vs. private facilities are aggregated by region only due to small sample sizes in some of the included countries.

## 3 RESULTS

The analysis included 194,042 children born in the 24 months preceding the survey. Overall, about half of births took place at home and half at a facility (52.3% and 47.7%, respectively; Table 2). There was considerable variation by region, with a higher prevalence of facility births in both the Middle East/Europe and Latin America (79.1% and 75.2%, respectively). Among home deliveries, the percentage of births assisted by a SBA ranged from around 1 in 20 to 1 in 4, and was lowest in Sub‐Saharan Africa and highest in Middle East/Europe. In most regions, deliveries at public facilities outnumbered those at private facilities. The exception was South/Southeast Asia, where there was a higher proportion of deliveries in private sector facilities.

**Table 2:** Percentages of births in sample by childbirth location, by region and overall for 57 included countries (*n* = 194,042)

Childbirth location	Sub‐Saharan Africa	Middle East/Europe	South/Southeast Asia	Latin America	Weighted mean of regions
Home	53.1	20.9	57.0	24.8	52.3
Without SBA	50.3	16.0	49.1	22.0	46.4
With SBA	2.8	4.9	7.9	2.8	5.9
Facility	46.9	79.1	43.0	75.2	47.7
Public sector	36.5	50.2	19.0	68.4	28.3
Private sector	10.4	28.9	24.0	6.8	19.4
Total	100.0	100.0	100.0	100.0	100.0

*Note*. SBA = skilled birth attendant.

**Figure 1:**
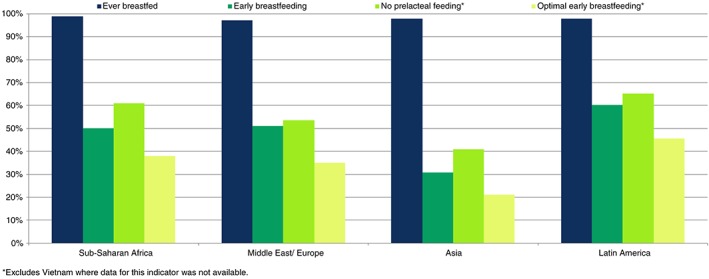
Breastfeeding indicators for all children (irrespective of childbirth location) by region

Figure 1 presents early breastfeeding indicators for all children by region. The percentage of children ever‐breastfed was consistently high across all regions (weighted mean 98.2%, range of countries 87.8–99.8%; Table 3) and across all childbirth locations, and this indicator is therefore not reported further.

**Table 3:** Summary of early breastfeeding indicators (percentage) by childbirth location, with heatmap shading^1^

	Sub‐Saharan Africa	Middle East/ Europe	Asia	Latin America	Weighted mean of regions	Median (range) in countries^3^
**Ever breastfed**	98.8%	97.1%	97.9%	97.9%	98.2%	98.4% (87.8%,99.8%)
Home	98.9%	98.7%	98.6%	99.0%	98.7%	99.0% (91.1%,100.0%)
Without SBA	98.9%	98.8%	98.7%	99.0%	98.8%	99.0% (87.7%,100.0%)
With SBA	99.2%	98.2%	97.4%	98.7%	97.8%	98.5% (92.3%,100.0%)
Facility	98.8%	96.6%	97.1%	97.5%	97.6%	98.0% (87.1%,99.8%)
Public sector	98.8%	96.3%	97.8%	97.7%	98.0%	98.1% (87.1%,99.8%)
Private sector	98.6%	97.2%	96.6%	95.6%	97.0%	97.4% (83.2%,100.0%)
**Early breastfeeding**	50.0%	51.0%	30.8%	60.3%	39.3%	52.5% (23.6%,95.6%)
Home	45.3%	61.5%	27.1%	57.6%	34.5%	50.3% (17.2%,95.5%)
Without SBA	45.0%	59.4%	26.5%	56.0%	34.2%	50.5% (16.6%,95.5%)
With SBA	50.1%	68.4%	31.3%	70.4%	36.9%	49.0% (21.5%,81.5%)
Facility	55.4%	48.3%	35.6%	61.2%	44.6%	55.5% (26.1%,95.6%)
Public sector	56.9%	49.0%	40.7%	62.8%	50.0%	55.1% (26.6%,95.2%)
Private sector	50.2%	46.9%	31.6%	45.2%	36.6%	49.2% (21.7%,97.0%)
**No prelacteal feeding** 2	61.0%	53.7%	41.0%	65.2%	49.2%	69.3% (2.7%,96.8%)
Home	51.6%	52.2%	35.4%	73.0%	41.7%	61.2% (2.6%,94.9%)
Without SBA	51.3%	49.2%	35.2%	73.6%	41.8%	61.4% (2.5%,94.9%)
With SBA	56.4%	62.2%	36.4%	68.1%	41.4%	52.2% (3.1%,85.8%)
Facility	71.7%	54.0%	48.9%	62.7%	57.7%	72.4% (3.5%,97.3%)
Public sector	72.9%	58.0%	58.7%	64.3%	65.3%	73.5% (3.2%,97.7%)
Private sector	67.4%	47.2%	42.2%	46.1%	47.2%	64.2% (15.3%,95.9%)
**Optimal early breastfeeding** 2	37.9%	35.0%	21.1%	45.6%	28.4%	39.2% (0.1%,93.2%)
Home	31.4%	39.3%	17.9%	46.8%	23.4%	39.6% (0.1%,91.5%)
Without SBA	31.3%	36.3%	17.6%	46.1%	23.3%	37.3% (0.1%,91.5%)
With SBA	34.3%	49.0%	19.8%	52.9%	24.3%	30.6% (0.0%,74.5%)
Facility	45.2%	33.8%	25.7%	45.2%	34.0%	43.7% (0.2%,93.6%)
Public sector	46.7%	35.3%	30.8%	46.8%	39.6%	43.4% (0.2%,93.5%)
Private sector	39.9%	31.2%	22.2%	29.3%	26.3%	39.3% (6.9%,93.9%)

*Note*. SBA = skilled birth attendant.

1 Shading indicates the most favourable (darkest) childbirth location sub‐type through to the least favourable (lightest), stratified by region and breastfeeding indicator.

2 Excludes Vietnam where data for this indicator were not available.

3 Excludes countries where was <50 observations in a category.

Rates of early initiation of breastfeeding and avoidance of prelacteal feeding were substantially lower (weighted mean 39.3% and 49.2%; median for countries 52.5% and 69.3%). At the region level, the highest prevalence of both was in Latin America (60.3% early breastfeeding, 65.2% no prelacteal feeding) and the lowest was South/Southeast Asia (30.8% and 41.0%, respectively). Across all regions, 28.4% (weighted mean) of children experienced both early breastfeeding initiation and avoidance of prelacteal feeding—defined as optimal early breastfeeding (country median 39.2%). The proportion of children benefiting from optimal early breastfeeding was highest in Latin America (45.6%) and lowest in South/Southeast Asia (21.1%).

### 3.1 Home vs. facility deliveries

**Figure 2:**
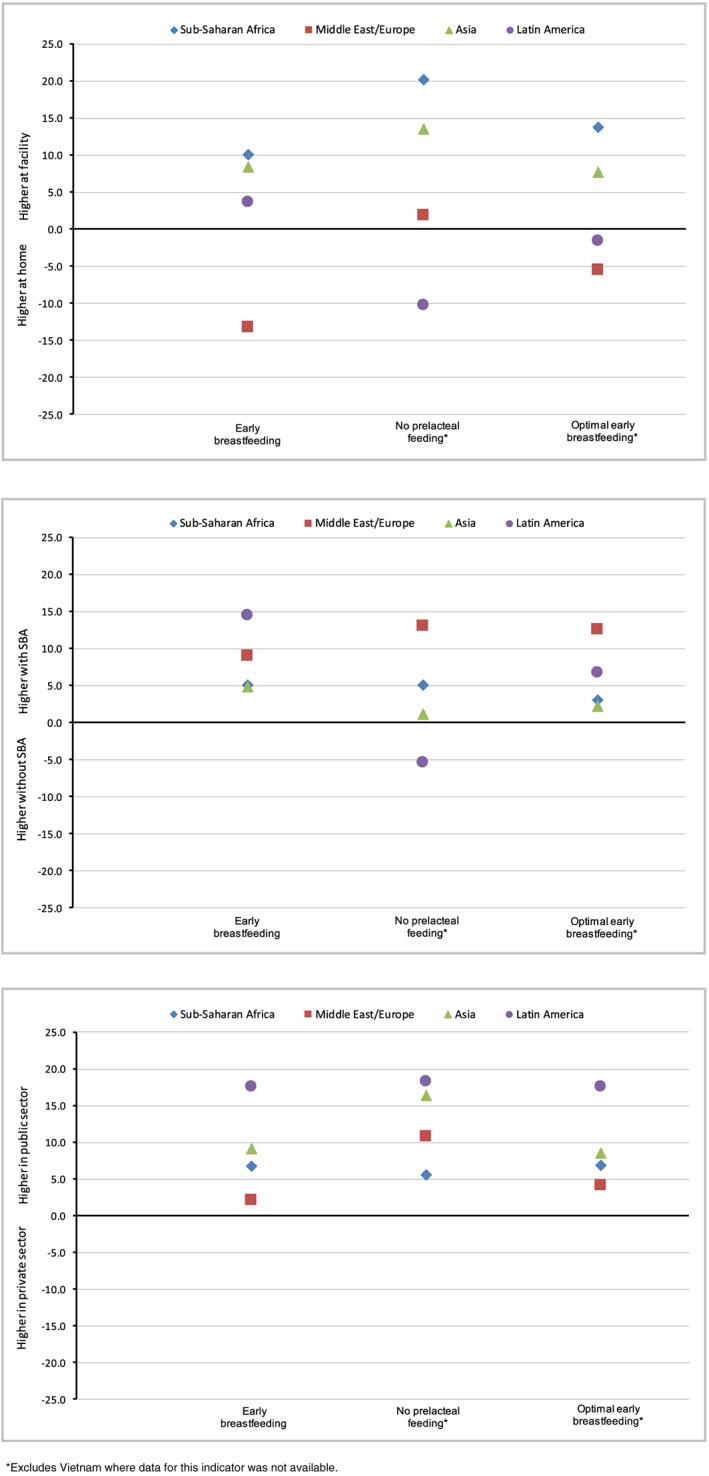
(a) Percent difference in breastfeeding indicators between home and facility births, region‐level data; (b) percent difference in breastfeeding indicators between SBA and non‐SBA attended home births, region‐level data; and (c) percent difference in breastfeeding indicators between private and public sector among facility births, region‐level data

**Figure 3:**
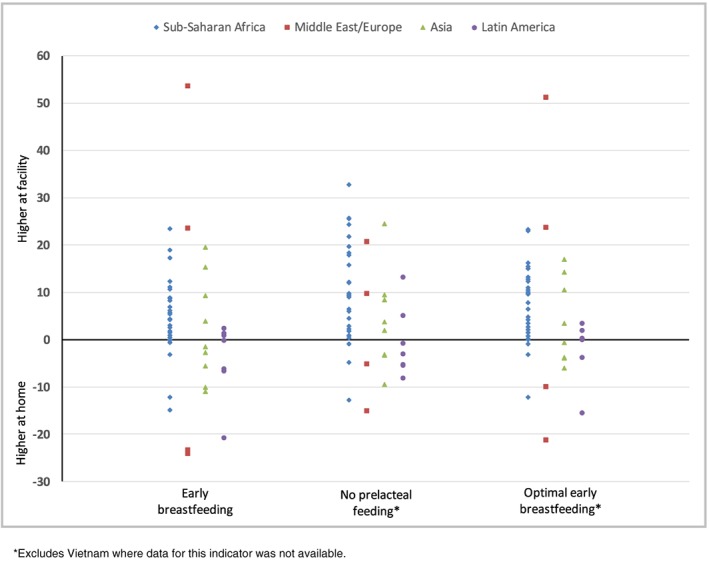
Percent difference in breastfeeding indicators between home and facility births, country‐level data by region

In comparing of home and facility childbirth locations, early breastfeeding outcomes were generally more favourable among facility births compared to home births (early initiation 44.6% vs. 34.5%, no prelacteal feeding 57.7% vs. 41.7%, early optimal breastfeeding 34.0% vs. 23.4%; Table 3). At the region level, early breastfeeding initiation was more favourable among facility births in all regions other than Middle East/Europe, and avoidance of prelacteal feeding was also higher among facility births in all regions other than Latin America (Figure 2a). Optimal early breastfeeding was higher among facility births compared to home births in Sub‐Saharan Africa and South/Southeast Asia, but not in Latin American and the Middle East/Europe. The largest and most consistent relative difference in outcomes between home vs. facility deliveries was observed for Sub‐Saharan Africa, where virtually all countries reported more favourable outcomes for facility births. This trend is emphasised by the country‐data points for this cross‐sectoral comparison presented in Figure 3.

### 3.2 Home deliveries with SBA vs. without SBA

Compared to home deliveries without SBA, early initiation of breastfeeding was slightly higher for births assisted by a SBA overall (36.9% with SBA vs. 34.2% without SBA) and at the region level. The prevalence of no prelacteal feeding showed little variation by the presence of a SBA across all regions (mean 41.4% with SBA, 41.8% without SBA), but region‐specific comparisons show that no prelacteal feeding was higher among home births attended by a SBA in all regions other than Latin America (Figure 2b). Optimal early breastfeeding was slightly more prevalent among home deliveries with a SBA compared to those without (24.3% vs. 23.3%).

### 3.3 Public sector deliveries vs. private sector deliveries

Among facility deliveries, all early breastfeeding outcomes were more favourable for deliveries in public sector facilities compared to private sector facilities (Figure 2c). The mean early initiation rate for public sector facilities was 50.0% compared to 36.6% in private sector deliveries (Table 3). The avoidance of prelacteal feeding was higher among public sector deliveries (65.3% vs. 47.2% for private sector deliveries), as was optimal early breastfeeding (39.6% and 26.3% for public and private facility deliveries respectively). These patterns were consistent across all regions, with the largest absolute differences between public sector and private sector deliveries observed in Latin America.

## 4 DISCUSSION

Using data from nationally representative surveys in 57 LMICs, we compared early breastfeeding outcomes by childbirth location. We found that early breastfeeding outcomes (early initiation, no prelacteal feeding, and a combined outcome “optimal breastfeeding”) were generally more favourable for facility births compared to home births, particularly in Sub‐Saharan Africa and South/Southeast Asia. Within home deliveries, early breastfeeding outcomes were more favourable among deliveries attended by a SBA, with the exception of prelacteal feeding avoidance in Latin America. The prevalence of positive early breastfeeding practices was higher among public sector births compared to private sector births in all four regions.

#### Strengths and limitations

This study is the most comprehensive assessment to date of early breastfeeding practices by childbirth setting. Our analysis was based on a large sample: nearly 200,000 births across 57 LMICs, with data taken from the most recent nationally‐representative surveys (Measure DHS) conducted since 2000.

Data on breastfeeding were collected retrospectively. A review of studies assessing the validity and reliability of maternal recall in relation to a range of breastfeeding indicators suggested that in general short‐term recall of breastfeeding is reliable, particularly when the recall period is 3 years or less (Li, Scanlon, & Serdula, 2005), as in our analysis (recall period 24 months). However, this review did not include any studies specifically assessing early breastfeeding practices. Measuring breastfeeding is challenging, and even the use of standardised questions may be interpreted differently according to the sociocultural context and the use of probing questions by interviewers (Salasibew, Filteau, & Marchant, 2014).

In interpreting region‐level totals, it is important to note that over 80% of the population in Sub‐Saharan Africa and South/Southeast Asia regions were represented by a survey in the analysis, but proportionally fewer country data points were recorded for Latin America and the Middle East/Europe (approximately one‐third regional population coverage). A further limitation of our analysis is that when stratifying by setting subtype (i.e., home births with and without a SBA; public vs. private facility deliveries), small numbers resulted in some extreme, potentially misleading values. For this reason, we avoided presenting country level data for comparisons other than home vs. facility births.

We included breastfeeding practices relating to the most recent birth in the 24 month period. This use of the most recent birth would result in the under‐representation of children born to higher‐fertility women, with these women likely to differ from lower‐fertility women in terms of sociodemographic characteristics. However, few women had more than one birth in the preceding 24 months (0.7–6.7% across each of the 57 countries).

The classification of childbirth locations into public (governmental) and private (all others) facilities was based on DHS response options presented on surveys. Due to the survey limitations, we were unable to further stratify private facilities into for‐profit and not‐for‐profit, or separately consider NGO and faith‐based providers (Blanc, Diaz, McCarthy, & Berdichevsky, 2016; Footman et al., 2015). We also note the limitation in women's ability to recall and correctly report the level of skill for her birth attendant, which is relevant for our categorisation of home deliveries. We attempted to avoid some of the issues related to recall by only including each woman's most recent births in the 2‐year period prior to survey.

The results presented here are descriptive only. Where a choice of childbirth setting is available, the individual characteristics of service users may explain differences in breastfeeding practice by delivery setting. Compared to poorer women, a higher proportion of richer women receive appropriate delivery care (Benova et al., 2015). Although there is little consistent evidence that early breastfeeding practices differ by indicators of wealth (Barros, Victora, Scherpbier, & Gwatkin, 2010), adjusting for socioeconomic characteristics would be desirable. In a recent analysis of data from the WHO Global Survey on Maternal and Perinatal Health, early initiation of breastfeeding was lower in private sector facilities compared to public sector facilitates. After adjusting for potential confounding, this difference was no longer significant (Takahashi et al., 2017).

#### Interpretation

In general, the trend for breastfeeding outcomes to be more favourable among appropriate childbirth care settings was strongest and most consistent in Sub‐Saharan Africa and South/Southeast Asia. It is possible that the weaker relationship between childbirth location in the mostly middle‐income regions of Middle East/Europe and Latin America may be partially explained by more favourable breastfeeding practices irrespective of childbirth setting; for example, early initiation of breastfeeding was twice as high in Latin America compared to South/Southeast Asia (60.3% vs 30.8%). Specific components of delivery care which are rapidly increasing in many middle‐income countries, such as increased use of analgesia, labour induction and augmentation, and caesarean delivery, may negatively impact on breastfeeding and contribute to a narrowing of the advantage conferred by facility care. An investigation of delivery setting and essential newborn care using data from prospective trials in South Asia reported that the proportion of women experiencing skin‐to‐skin contact with their infant within 30 min of birth was lower in institutional delivery settings compared to home (Pagel et al., 2014). Among facility births, we consistently observed a higher prevalence of optimal early breastfeeding practices in public sector births compared to births in private facilities. Differences in early breastfeeding practices by sector of facility were largest in Latin America; between 15% and 20% higher in for births in the public sector for all breastfeeding outcomes. In a recent assessment of childbirth location using DHS data, 24% of all deliveries in Latin America were by caesarean section, rising to 45% for births in the private sector (Benova et al., 2015). The high prevalence of caesarean delivery, known to be associated with lower breastfeeding initiation (Dewey, Nommsen‐Rivers, Heinig, & Cohen, 2003; Miller et al., 2016; Takahashi et al., 2017; Zanardo et al., 2010), may help to explain the public–private differential in this region. Quality of care may also differ by sector. In data from the WHO Global Survey on Maternal and Perinatal Health, facilities reporting the availability of guidelines for post‐natal and/or neonatal care (taken as a proxy for quality of care) reported an early initiation rate twice as high as facilities with no such guidelines (Takahashi et al., 2017).

Compared to women who deliver at home, women who give birth in facilities tend to be wealthier (Campbell et al., 2015). Victora et al. (2016) report that wealthier women in LMICs are increasingly adopting exclusive breastfeeding at a faster rate than women from poorer backgrounds. Increasing uptake of skilled childbirth care among socioeconomically advantaged women may contribute to this trend, exacerbating existing inequalities in child health outcomes by wealth quintile.

Given the probable socioeconomic differences between women giving birth in different settings, future research should adjust for these and other characteristics in an attempt to assess the independent role of childbirth location on breastfeeding practices. Further information on the characteristics of different facility settings in terms of staffing, resources, and institutional practices may also help to explain the observed differences between breastfeeding practices in the public and private sector.

## 5 CONCLUSION

Low‐income countries, such as the majority of those included in the South/Southeast Asian and Sub‐Saharan Africa grouping used here, clearly have the most to gain from improvements in breastfeeding. In their recent review of breastfeeding in the 21st century, Victora et al. (2016) note that compared to HICs, LMICs often have better data on breastfeeding. The analysis reported here is an attempt to utilise standardised data to address a knowledge gap concerning the relationship between childbirth location and breastfeeding. Our analysis demonstrates that while breastfeeding initiation is near‐universal in LMICs, rates of optimal early breastfeeding are less satisfactory and show considerable variation by childbirth location. The benefits of appropriate delivery care are often judged in relation to immediate maternal and infant outcomes. Childbirth care with elements of higher‐level skill or enabling environment, particularly care delivered in public sector facilities—also appears to be positively correlated with optimal breastfeeding.

## CONFLICTS OF INTEREST

The authors declare that they have no conflicts of interest.

## CONTRIBUTIONS

LB, DM, and LO conceived and designed the study. DM prepared the data, and LB and DM analysed the data. All authors interpreted the results. LO wrote the first draft of the manuscript, LB critically revised the manuscript. All authors contributed to and approved the final manuscript. OC provided overall guidance.

## Supporting information

Table S1.
Early breastfeeding indicators (percentage) by country and childbirth location*

## References

[mcn12535-bib-0001] Akuse, R. M. , & Obinya, E. A. (2002). Why healthcare workers give prelacteal feeds. European Journal of Clinical Nutrition, 56, 729–734. 12122548 10.1038/sj.ejcn.1601385

[mcn12535-bib-0002] Barros, F. C. , Victora, C. G. , Scherpbier, R. , & Gwatkin, D. (2010). Socioeconomic inequities in the health and nutrition of children in low/middle income countries. Revista de Saúde Pública, 44, 1–16. 20140324 10.1590/s0034-89102010000100001

[mcn12535-bib-0003] Benova, L. , Macleod, D. , Footman, K. , Cavallaro, F. , Lynch, C. A. , & Campbell, O. M. (2015). Role of the private sector in childbirth care: Cross‐sectional survey evidence from 57 low‐and middle‐income countries using Demographic and Health Surveys. Tropical Medicine & International Health, 20, 1657–1673. 26412496 10.1111/tmi.12598

[mcn12535-bib-0004] Black, R. E. , Victora, C. G. , Walker, S. P. , Bhutta, Z. A. , Christian, P. , de Onis, M. , … Martorell, R. (2013). Maternal and child undernutrition and overweight in low‐income and middle‐income countries. The Lancet, 382, 427–451. 10.1016/S0140-6736(13)60937-X 23746772

[mcn12535-bib-0005] Blanc, A. K. , Diaz, C. , McCarthy, K. J. , & Berdichevsky, K. (2016). Measuring progress in maternal and newborn health care in Mexico: Validating indicators of health system contact and quality of care. BMC Pregnancy and Childbirth, 16, 255. 27577266 10.1186/s12884-016-1047-0 PMC5006493

[mcn12535-bib-0006] Campbell, O. M. , Benova, L. , Macleod, D. , Goodman, C. , Footman, K. , Pereira, A. L. , … Lynch, C. A. (2015). Who, What, Where: An analysis of private sector family planning provision in 57 low‐and middle‐income countries. Tropical Medicine & International Health, 20, 1639–1656. 26412363 10.1111/tmi.12597

[mcn12535-bib-0007] Chandrashekhar, T. S. , Joshi, H. S. , Binu, V. S. , Shankar, P. R. , Rana, M. S. , & Ramachandran, U. (2007). Breast‐feeding initiation and determinants of exclusive breast‐feeding—A questionnaire survey in an urban population of western Nepal. Public Health Nutrition, 10, 192–197. 17261229 10.1017/S1368980007248475

[mcn12535-bib-0008] Debes, A. K. , Kohli, A. , Walker, N. , Edmond, K. , & Mullany, L. C. (2013). Time to initiation of breastfeeding and neonatal mortality and morbidity: A systematic review. BMC Public Health, 13, S19. 24564770 10.1186/1471-2458-13-S3-S19 PMC3847227

[mcn12535-bib-0009] Dewey, K. G. , Nommsen‐Rivers, L. A. , Heinig, M. J. , & Cohen, R. J. (2003). Risk factors for suboptimal infant breastfeeding behavior, delayed onset of lactation, and excess neonatal weight loss. Pediatrics, 112, 607–619. 12949292 10.1542/peds.112.3.607

[mcn12535-bib-0010] Engebretsen, I. M. S. , Wamani, H. , Karamagi, C. , Semiyaga, N. , Tumwine, J. , & Tylleskär, T. (2007). Low adherence to exclusive breastfeeding in Eastern Uganda: A community‐based cross‐sectional study comparing dietary recall since birth with 24‐hour recall. BMC Pediatrics, 7, 10. 17331251 10.1186/1471-2431-7-10 PMC1828054

[mcn12535-bib-0011] Fabic, M. S. , Choi, Y. , & Bird, S. (2012). A systematic review of Demographic and Health Surveys: Data availability and utilization for research. Bulletin of the World Health Organization, 90, 604–612. 22893744 10.2471/BLT.11.095513 PMC3417790

[mcn12535-bib-0012] Fink, G. , Ross, R. , & Hill, K. (2015). Institutional deliveries weakly associated with improved neonatal survival in developing countries: Evidence from 192 Demographic and Health Surveys. International Journal of Epidemiology, 44, 1879–1888. 26130739 10.1093/ije/dyv115

[mcn12535-bib-0013] Footman, K. , Benova, L. , Goodman, C. , Macleod, D. , Lynch, C. , Penn‐Kekana, L. , & Campbell, O. M. (2015). Using multi‐country household surveys to understand who provides reproductive and maternal health services in low‐and middle‐income countries: A critical appraisal of the Demographic and Health Surveys. Tropical Medicine & International Health, 20, 589–606. 25641212 10.1111/tmi.12471 PMC4409817

[mcn12535-bib-0014] Khanal, V. , Adhikari, M. , Sauer, K. , & Zhao, Y. (2013). Factors associated with the introduction of prelacteal feeds in Nepal: Findings from the Nepal Demographic and Health Survey 2011. International Breastfeeding Journal, 8, 9. 23924230 10.1186/1746-4358-8-9 PMC3750657

[mcn12535-bib-0015] Kimani‐Murage, E. W. , Madise, N. J. , Fotso, J.‐C. , Kyobutungi, C. , Mutua, M. K. , Gitau, T. M. , & Yatich, N. (2011). Patterns and determinants of breastfeeding and complementary feeding practices in urban informal settlements, Nairobi Kenya. BMC Public Health, 11, 396. 21615957 10.1186/1471-2458-11-396 PMC3118248

[mcn12535-bib-0016] Li, R. , Scanlon, K. S. , & Serdula, M. K. (2005). The validity and reliability of maternal recall of breastfeeding practice. Nutrition Reviews, 63, 103–110. 15869124 10.1111/j.1753-4887.2005.tb00128.x

[mcn12535-bib-0017] Lim, S. S. , Vos, T. , Flaxman, A. D. , Danaei, G. , Shibuya, K. , Adair‐Rohani, H. , … Memish, Z. A. (2012). A comparative risk assessment of burden of disease and injury attributable to 67 risk factors and risk factor clusters in 21 regions, 1990–2010: A systematic analysis for the Global Burden of Disease Study 2010. Lancet, 380, 2224–2260. 23245609 10.1016/S0140-6736(12)61766-8 PMC4156511

[mcn12535-bib-0018] Miller, S. , Abalos, E. , Chamillard, M. , Ciapponi, A. , Colaci, D. , Comandé, D. , … Althabe, F. (2016). Beyond too little, too late and too much, too soon: A pathway towards evidence‐based, respectful maternity care worldwide. The Lancet. 10.1016/S0140-6736(16)31472-6 27642019

[mcn12535-bib-0019] Montagu, D. , Yamey, G. , Visconti, A. , Harding, A. , & Yoong, J. (2011). Where do poor women in developing countries give birth? A multi‐country analysis of demographic and health survey data. PLoS One, 6, e17155. 10.1371/journal.pone.0017155 PMC3046115 21386886

[mcn12535-bib-0020] Moore, E. R. , Anderson, G. C. , Bergman, N. , & Dowswell, T. (2012). Early skin‐to‐skin contact for mothers and their healthy newborn infants. Cochrane Database of Systematic Reviews. 10.1002/14651858.CD003519.pub3 PMC3979156 22592691

[mcn12535-bib-0021] Neovita Study Group . (2016). Timing of initiation, patterns of breastfeeding, and infant survival: Prospective analysis of pooled data from three randomised trials. The Lancet Global Health, 4, e266–e275. 27013313 10.1016/S2214-109X(16)00040-1

[mcn12535-bib-0022] Ogunlesi, T. A. (2010). Maternal socio‐demographic factors influencing the initiation and exclusivity of breastfeeding in a Nigerian semi‐urban setting. Maternal and Child Health Journal, 14, 459–465. 19156508 10.1007/s10995-008-0440-3

[mcn12535-bib-0023] Pagel, C. , Prost, A. , Hossen, M. , Azad, K. , Kuddus, A. , Roy, S. S. , … Crowe, S. (2014). Is essential newborn care provided by institutions and after home births? Analysis of prospective data from community trials in rural South Asia. BMC Pregnancy and Childbirth, 14, 1. 24606612 10.1186/1471-2393-14-99 PMC4016384

[mcn12535-bib-0024] Patel, A. , Banerjee, A. , & Kaletwad, A. (2013). Factors associated with prelacteal feeding and timely initiation of breastfeeding in hospital‐delivered infants in India. Journal of Human Lactation, 29, 572–578. 23427115 10.1177/0890334412474718

[mcn12535-bib-0025] Patil, C. L. , Turab, A. , Ambikapathi, R. , Nesamvuni, C. , Chandyo, R. K. , Bose, A. , … MAL‐ED network . (2015). Early interruption of exclusive breastfeeding: Results from the eight‐country MAL‐ED study. Journal of Health, Population and Nutrition, 34, 10. 10.1186/s41043-015-0004-2 PMC5025973 26825923

[mcn12535-bib-0026] Salasibew, M. M. , Filteau, S. , & Marchant, T. (2014). Measurement of breastfeeding initiation: Ethiopian mothers' perception about survey questions assessing early initiation of breastfeeding. International Breastfeeding Journal, 9, 13. 25180042 10.1186/1746-4358-9-13 PMC4150427

[mcn12535-bib-0027] Senarath, U. , Siriwardena, I. , Godakandage, S. S. , Jayawickrama, H. , Fernando, D. N. , & Dibley, M. J. (2012). Determinants of breastfeeding practices: An analysis of the Sri Lanka Demographic and Health Survey 2006–2007. Maternal & Child Nutrition, 8, 315–329. 21507202 10.1111/j.1740-8709.2011.00321.x PMC6860852

[mcn12535-bib-0028] Shirima, R. , Greiner, T. , Kylberg, E. , & Gebre‐Medhin, M. (2001). Exclusive breast‐feeding is rarely practised in rural and urban Morogoro, Tanzania. Public Health Nutrition, 4. 10.1079/phn200057 11299086

[mcn12535-bib-0029] Sundaram, M. E. , Labrique, A. B. , Mehra, S. , Ali, H. , Shamim, A. A. , Klemm, R. D. W. , … Christian, P. (2013). Early neonatal feeding is common and associated with subsequent breastfeeding behavior in rural Bangladesh. The Journal of Nutrition, 143, 1161–1167. 23677862 10.3945/jn.112.170803

[mcn12535-bib-0030] Takahashi, K. , Ganchimeg, T. , Ota, E. , Vogel, J. P. , Souza, J. P. , Laopaiboon, M. , … Mori, R. (2017). Prevalence of early initiation of breastfeeding and determinants of delayed initiation of breastfeeding: Secondary analysis of the WHO Global Survey. Scientific Reports, 7, 44868. 28322265 10.1038/srep44868 PMC5359598

[mcn12535-bib-0031] Unicef . (2016). The state of the world's children 2016: A fair chance for every child. New York: United National Children's Fund (UNICEF).

[mcn12535-bib-0032] Victora, C. G. , Bahl, R. , Barros, A. J. D. , França, G. V. A. , Horton, S. , Krasevec, J. , … Lancet Breastfeeding Series Group . (2016). Breastfeeding in the 21st century: Epidemiology, mechanisms, and lifelong effect. The Lancet, 387, 475–490. 10.1016/S0140-6736(15)01024-7 26869575

[mcn12535-bib-0033] World Health Organization . (2008). Indicators for assessing infant and young child feeding practices: Part 1 ‐ Definitions. Washington DC [USA]: World Health Organization. Dept. of Child and Adolescent Health and Development.

[mcn12535-bib-0034] Zanardo, V. , Svegliado, G. , Cavallin, F. , Giustardi, A. , Cosmi, E. , Litta, P. , & Trevisanuto, D. (2010). Elective cesarean delivery: Does it have a negative effect on breastfeeding? Birth, 37, 275–279. 21083718 10.1111/j.1523-536X.2010.00421.x

