# Supplementary material for: Early breastfeeding practices: Descriptive analysis of recent Demographic and Health Surveys
Source: Matern Child Nutr. 2017 Oct 16;14(2):e12535. doi: 10.1111/mcn.12535 (PMC5900960; doi:10.1111/mcn.12535)
Supplement: Supplementary file 1 — Table S1. Early breastfeeding indicators (percentage) by country and childbirth location* [file MCN-14-e12535-s001.pdf]

Table S1. Early breastfeeding indicators (percentage) by country and childbirth location*

Country	Year	Early breastfeeding initiation			No prelacteal breastfeeding			Optimal early breastfeeding		
		All	Home	Facility	All	Home	Facility	All	Home	Facility
Benin	2006	54.67%	39.34%	58.27%	75.40%	55.71%	80.03%	47.54%	28.67%	51.97%
Burkina Faso	2010	42.35%	35.77%	44.67%	63.32%	44.27%	70.04%	29.86%	17.85%	34.09%
Burundi	2010	74.76%	74.13%	75.03%	92.35%	92.97%	92.08%	71.32%	70.26%	71.77%
Cameroon	2011	40.45%	35.19%	43.52%	46.53%	25.86%	58.64%	26.18%	16.69%	31.74%
Chad	2004	32.62%	32.40%	33.95%	2.71%	2.57%	3.54%	0.10%	0.09%	0.18%
Congo-B	2005	34.84%	33.32%	35.17%	62.27%	49.18%	65.02%	28.18%	22.81%	29.31%
DRC	2007	48.63%	47.96%	48.89%	83.03%	86.53%	81.70%	44.43%	45.08%	44.18%
Ethiopia	2011	52.50%	52.21%	54.80%	72.00%	71.26%	77.71%	44.23%	44.15%	44.90%
Gabon	2012	33.59%	47.46%	32.53%	52.28%	64.22%	51.37%	22.69%	33.96%	21.83%
Ghana	2008	52.66%	48.60%	55.50%	82.10%	80.40%	83.28%	46.76%	42.17%	49.98%
Guinea	2005	38.29%	37.03%	41.33%	27.00%	22.38%	38.17%	17.12%	14.03%	24.60%
Kenya	2008-9	56.90%	55.51%	58.57%	57.09%	47.13%	69.01%	38.52%	33.52%	44.51%
Lesotho	2009	51.77%	49.14%	53.38%	68.23%	55.99%	75.68%	40.37%	32.41%	45.22%
Liberia	2007	66.84%	71.76%	59.54%	74.45%	73.72%	75.53%	52.81%	54.06%	50.95%
Madagascar	2008-9	71.44%	69.92%	74.27%	73.96%	70.66%	80.10%	60.10%	57.35%	65.21%
Malawi	2010	95.56%	95.50%	95.58%	96.79%	94.93%	97.26%	93.20%	91.54%	93.62%
Mali	2006	44.82%	39.38%	50.52%	52.26%	43.34%	61.64%	31.70%	26.81%	36.83%
Mozambique	2011	77.64%	77.75%	77.56%	92.94%	92.45%	93.27%	74.84%	74.83%	74.85%
Namibia	2006-7	68.06%	67.71%	68.14%	80.65%	75.70%	81.76%	60.38%	57.58%	61.00%
Niger	2006	46.75%	43.52%	60.80%	19.51%	17.26%	29.32%	15.94%	14.15%	23.73%
Nigeria	2008	37.38%	33.50%	44.18%	42.08%	32.81%	58.36%	22.74%	17.93%	31.19%
Rwanda	2010	72.54%	65.21%	74.14%	85.66%	75.64%	87.86%	66.20%	56.13%	68.41%
Sao Tome and Principe	2008-9	43.92%	44.35%	43.81%	86.45%	86.23%	86.50%	42.66%	42.09%	42.80%
Senegal	2010-11	48.80%	42.38%	51.16%	46.03%	43.91%	46.80%	29.81%	26.78%	30.93%
Sierra Leone	2008	49.42%	50.23%	47.00%	38.50%	36.25%	45.31%	22.78%	22.11%	24.82%
Swaziland	2006-7	59.14%	54.84%	60.51%	75.75%	68.30%	78.12%	51.57%	44.13%	53.94%
Tanzania	2010	46.07%	33.89%	57.39%	67.88%	58.63%	76.47%	36.97%	25.08%	48.02%
Uganda	2011	53.36%	50.10%	55.52%	58.05%	55.35%	59.84%	34.85%	32.77%	36.23%
Zambia	2007	56.48%	53.52%	59.76%	90.13%	90.58%	89.63%	53.20%	50.89%	55.76%
Zimbabwe	2010-11	66.93%	58.91%	71.27%	85.42%	79.39%	88.69%	59.21%	49.19%	64.65%
Albania	2008-9	43.51%		43.40%	79.35%		79.37%	38.67%		38.57%
Armenia	2010	35.97%		35.97%	87.34%		87.34%	34.24%		34.24%
Azerbaijan	2006	30.71%	49.85%	26.48%	52.42%	64.71%	49.70%	25.47%	42.87%	21.62%
Egypt	2008	54.23%		47.52%	51.10%		47.96%	36.84%		31.75%
Jordan	2007	37.24%		36.95%	38.61%		38.28%	21.50%		21.21%
Moldova	2005	66.44%	13.21%	66.93%	89.52%	79.86%	89.61%	64.09%	13.21%	64.55%
Morocco	2003-4	48.72%	64.27%	40.20%	32.69%	35.98%	30.89%	18.47%	24.90%	14.95%
Turkey	2003	52.59%	33.34%	56.98%	60.01%	43.16%	63.86%	39.74%	20.37%	44.16%
Ukraine	2007	41.45%		41.45%	74.55%		74.55%	32.98%		32.98%
Bangladesh	2011	47.11%	50.44%	39.45%	60.68%	59.56%	63.27%	34.50%	35.68%	31.79%

**Cambodia**	2010	**67.01%**	60.76%	70.10%	**78.88%**	77.57%	79.53%	**57.29%**	50.22%	60.78%
**India**	2005-6	**23.55%**	17.24%	32.64%	**41.78%**	31.75%	56.21%	**19.35%**	13.51%	27.76%
**Indonesia**	2007	**40.24%**	41.60%	38.91%	**32.09%**	36.88%	27.40%	**24.04%**	27.07%	21.08%
**Maldives**	2009	**60.54%**		60.24%	**84.49%**		84.38%	**54.65%**		54.34%
**Nepal**	2011	**45.46%**	36.70%	56.20%	**71.68%**	67.92%	76.29%	**37.39%**	29.73%	46.78%
**Pakistan**	2006-7	**27.03%**	27.60%	26.13%	**31.27%**	27.62%	37.06%	**13.53%**	12.18%	15.66%
**Philippines**	2008	**48.08%**	52.83%	42.79%	**41.91%**	43.37%	40.29%	**26.97%**	28.76%	24.98%
**Timor-Leste**	2009-10	**80.33%**	79.36%	83.27%	**86.42%**	87.23%	83.99%	**74.71%**	74.86%	74.22%
**Vietnam**	2002	**53.68%**	58.06%	52.52%	n/a	n/a	n/a	n/a	n/a	n/a
**Bolivia**	2008	**63.58%**	63.72%	63.53%	**71.79%**	75.49%	70.27%	**52.08%**	54.76%	50.98%
**Colombia**	2010	**63.94%**	70.32%	63.65%	**61.44%**	56.57%	61.66%	**45.28%**	45.28%	45.28%
**Dominican Republic**	2007	**61.47%**		61.32%	**39.81%**		39.40%	**34.74%**		34.40%
**Guyana**	2009	**61.41%**	59.21%	61.65%	**76.92%**	79.65%	76.63%	**54.59%**	54.34%	54.62%
**Haiti**	2012	**47.55%**	47.19%	48.08%	**78.59%**	78.90%	78.12%	**41.03%**	39.63%	43.15%
**Honduras**	2011-12	**64.59%**	82.34%	61.57%	**54.27%**	61.19%	53.09%	**42.09%**	55.30%	39.84%
**Nicaragua**	2001	**72.65%**	76.95%	70.85%	**72.49%**	63.14%	76.39%	**59.90%**	58.57%	60.46%
**Peru**	2000	**55.10%**	54.30%	55.69%	**70.41%**	73.56%	68.08%	**45.86%**	45.80%	45.91%

*****Blank cells indicate <50 observations
